# Evaluation of Retentive Forces in Three Types of Removable Partial Denture Framework Materials: An In Vitro Study

**DOI:** 10.7759/cureus.76269

**Published:** 2024-12-23

**Authors:** Ribaz Tahsin Kakai

**Affiliations:** 1 Dentistry, Kurdistan Higher Council of Medical Specialties, Erbil, IRQ

**Keywords:** computer-aided design/computer-aided manufacturing, kennedy class i, metal alloy, polyetherketonketone, retention force

## Abstract

Introduction

The utilization of Computer-Aided Design and Computer-Aided Manufacturing (CAD/CAM) technology in the production of polyetheretherketone (PEEK) and acetal frameworks enhances the precision and stability of partial denture frameworks. This study evaluates the retentive forces of CAD/CAM-fabricated PEEK, acetal, and cobalt-chromium (Co-Cr) frameworks in removable partial dentures (RPDs).

Methods

Forty-five frameworks were fabricated (15 each of PEEK, acetal, and Co-Cr) and tested for retentive forces using a universal testing machine at a crosshead speed of 5 mm/min. The frameworks were divided into three groups: one with digitally milled PEEK, one with Co-Cr, and one with digitally milled acetal. Each partial denture clasp was designed using CAD/CAM software, extending between the central incisor and the second premolar. These clasps were specifically designed to aid in securing the framework during tensile testing and to facilitate the removal of cast clasps during later stages.

Results

PEEK frameworks demonstrated the highest retentive forces (mean: 3.52 ± 0.78 N, p < 0.001), followed by acetal (mean: 0.47 ± 0.02 N) and Co-Cr (mean: 0.32 ± 0.12 N).

Conclusions

PEEK outperformed other materials, making it a promising candidate for RPD frameworks.

## Introduction

Removable partial dentures (RPDs) are widely used to address partial tooth loss, providing customizable solutions that meet diverse patient needs [1]. Despite their popularity, RPDs face unique challenges in cases such as Kennedy Class I distal extensions, where the absence of posterior teeth can result in reduced vertical occlusion, compromised stability, and accelerated wear of anterior teeth [2].

Since the advent of acrylic polymers and chrome cobalt alloys in dentistry several decades ago, RPDs have gained widespread popularity. Patients frequently choose these devices due to their affordability and compatibility with physiological needs. Recently, flexible partial dentures have emerged as a preferable alternative, offering enhanced comfort, durability, and long-term utility, leading to increased recommendations by dental professionals [3].

Traditional RPDs, while cost-effective and reliable for replacing missing teeth, are undergoing significant evolution with advancements in manufacturing technologies. The integration of Computer-Aided Design (CAD), Computer-Aided Manufacturing (CAM), and rapid prototyping methods has facilitated more precise and efficient production of RPDs [4].

Historically, metal alloys such as titanium and gold have been used to fabricate the metal frameworks of RPDs. However, clinical practice has increasingly turned to thermoplastic materials, responding to patient concerns regarding the metallic appearance, added weight, potential metallic taste, and risk of allergic reactions [5].

Among thermoplastic materials, polyoxymethylene (POM), commonly known as acetal, was introduced in 1971 as a robust and durable option for RPD frameworks. The early development of fast-injection technologies enabled the creation of tooth-colored clasps with thermoplastic fluoropolymers, enhancing aesthetic appeal [6].

Furthermore, advanced thermoplastic polymers such as polyetheretherketone (PEEK) and polyetherketoneketone (PEKK), members of the polyaryletherketone (PAEK) family, represent a new era in RPD materials. These polymers, characterized by high-temperature resistance and a stable molecular structure, provide a viable alternative to traditional metals in prosthodontics [7].

PEEK is particularly noted for its high melting point (343°C or 649°F) and exceptional mechanical properties, including tensile strength, stiffness, and thermal stability under high temperatures. Additionally, PEEK’s resistance to acids, bases, and organic solvents, alongside its biocompatibility, makes it suitable for demanding environments in medical and industrial applications. Consequently, it has been applied in fields ranging from spinal and dental implants to components in the aerospace and automotive industries [8,9].

In contrast, acetal (POM) is highly valued for its rigidity, dimensional stability, and low friction, making it ideal for precision components such as gears, bearings, and electrical insulators. Though acetal lacks the high-temperature resilience of PEEK, it remains resistant to a wide range of solvents and fuels, providing consistent performance in many industrial applications [10]. Overall, PEEK is preferred for chemically and thermally intense environments, whereas acetal excels in applications requiring low friction and precise machinability [11].

Despite the durability and biocompatibility of metal-based RPDs, traditional cobalt-chromium (Co/Cr) frameworks face aesthetic and hypersensitivity challenges, particularly for patients with sensitivity to metal. Polymer-based RPD frameworks offer aesthetic advantages, being transparent and color-matched, and are generally more lightweight and cost-effective than metal frameworks. Additionally, their lower water absorption and solubility contribute to increased longevity, while their elastic nature facilitates easier fabrication, repair, and replication [12]. This study aims to investigate and compare the retention properties of CAD/CAM-fabricated Co/Cr, acetal, and PEEK RPD frameworks.

## Materials and methods

Study design

**Figure 1:**
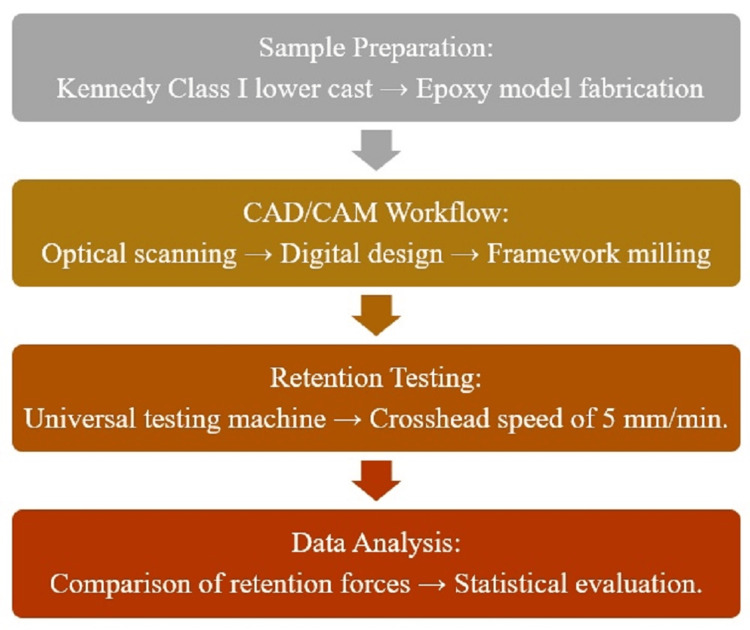
Study flowchart CAD/CAM: Computer-Aided Design and Computer-Aided Manufacturing

In this study, we employed an existing partially edentulous stone model to create a mandibular epoxy resin model, which was designed to reflect the characteristics of a Kennedy Class I situation. This model provided a reliable and consistent basis for our investigation, ensuring the accuracy of the findings. A flowchart summarizing the methodological steps of the study has been included (Figure 1). This diagram visually outlines the key processes, including sample preparation, CAD/CAM workflow, retention testing, and data analysis, ensuring a clear understanding of the research methodology.

Sample size determination

The sample size was calculated using Arkin's formula, which is widely applied in determining sample sizes for in-vitro studies. This approach considered a significance level of 0.05, a standard deviation of 4.55 (based on prior studies evaluating retention forces), and an absolute error margin of 2. Using a standard normal variate of 1.96, we determined that 45 samples would be sufficient for this study. These samples were evenly divided into three groups, ensuring balanced comparisons and statistical validity [13].

The crosshead speed of 5 mm/min was selected based on established protocols in dental research and its demonstrated reliability in similar studies of retentive forces [14]. This speed provides a controlled and consistent application of force, ensuring accurate and reproducible results during testing.

Grouping

**Figure 2:**
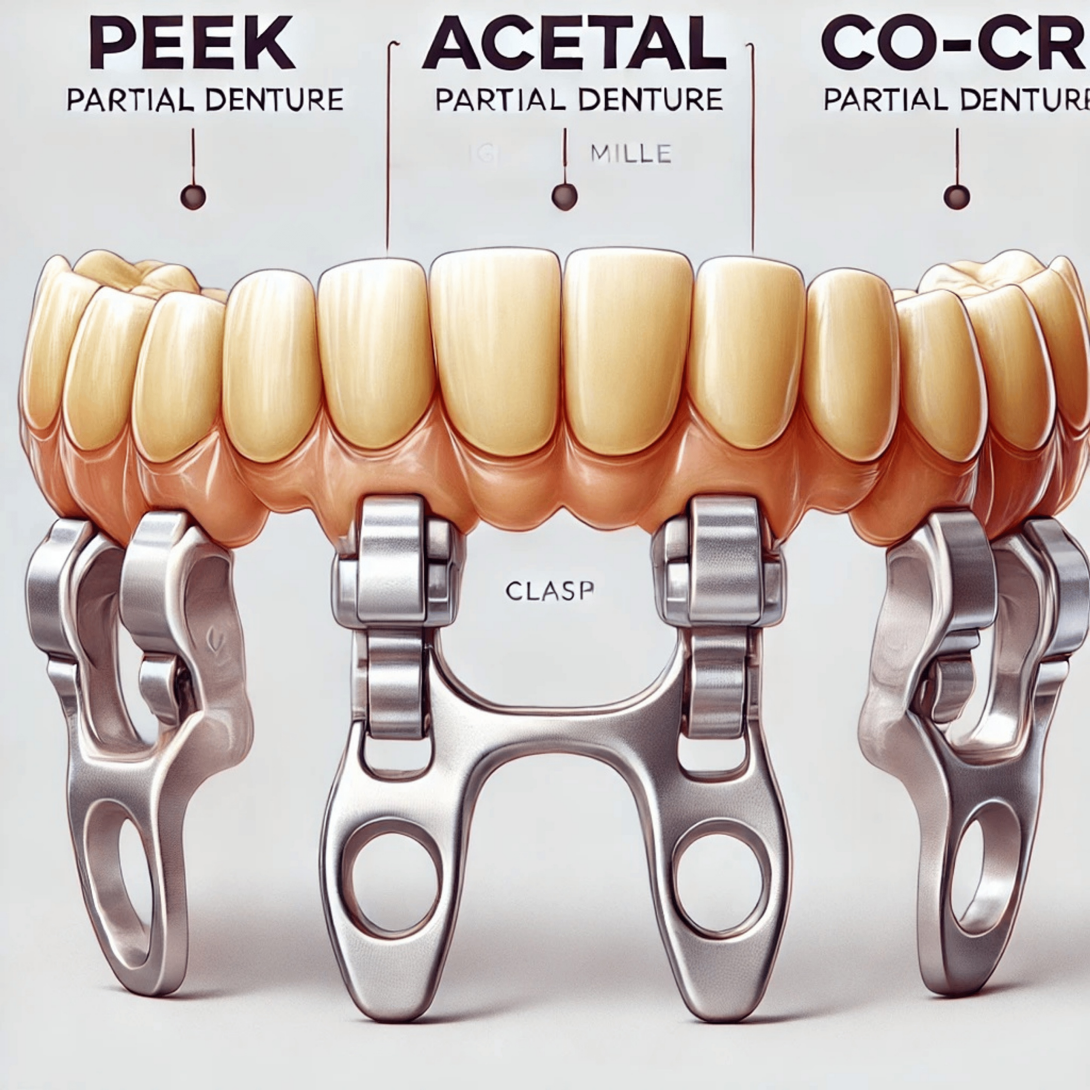
Samples of material types (PEEK, acetal, and Co-Cr) and their digitally milled designs Digitally milled partial denture frameworks were used in the study, showcasing the three material types: polyetheretherketone (PEEK), acetal, and cobalt-chromium (Co-Cr). The frameworks highlight the clasp design and dimensions, as prepared using Computer-Aided Design and Computer-Aided Manufacturing (CAD/CAM) technology.

Frameworks were assigned to one of three groups based on the type of framework: Group 1 (G I) consisted of 15 digitally milled PEEK frameworks, Group 2 (G II) included 15 digitally milled acetal frameworks, and Group 3 (G III) comprised 15 digitally milled Co-Cr frameworks. This grouping was essential for assessing the differences in retention among the various materials used. A representative figure of the digitally milled frameworks has been included (Figure 2) to illustrate the samples' design and material types. This visual representation complements the description of the methodology and helps clarify the experimental setup.

Fabrication of epoxy model and prosthetic procedures

The fabrication process began with the lower dental stone cast representing the Kennedy Class I classification. We created a negative impression using impression silicone, which was then filled with high-quality epoxy resin. The resulting epoxy model was carefully prepared to conform to the Kennedy Class I specifications. Additionally, we designed the prosthetic components, including rests, guide planes, and clasps, using advanced digital methods to achieve optimal fit and function.

Scanning and framework construction

The definitive cast was duplicated using silicone-based materials, allowing for the creation of an epoxy cast. This cast was then subjected to optical scanning, during which any undesirable undercuts were digitally blocked out. CAD/CAM software was utilized to design and fabricate the frameworks from Co-Cr, PEEK, and acetal. Each fabrication disc produced three frameworks, leading to a total of 15 frameworks for each material group.

Retention evaluation

The Instron universal testing machine (Instron Corporation, Norwood, Massachusetts, USA) was utilized to apply a tensile load at a crosshead speed of 5 mm/min until it reached an automated stop. This crosshead speed was selected based on established protocols in similar studies of dental retention testing, ensuring consistent application of force and reliable measurement of retentive forces [14]. Custom metal chains were attached to hooks on the frameworks, ensuring stability throughout the testing process. Each framework underwent vertical pulling from the epoxy cast at a consistent crosshead speed of 5 mm/min, and this procedure was repeated 10 times for each design [15]. Data were meticulously collected for subsequent analysis.

Statistical analysis

Statistical analyses were conducted to compare the retentive forces among the three material groups (PEEK, acetal, and Co-Cr) and identify significant differences. These methods were chosen to quantitatively evaluate the performance of each material, aligning with the study's objective of determining the most effective framework material. The analyses provided insights into the relative strengths of the materials, enabling evidence-based recommendations for clinical applications.

Ethical approval

This research was an in vitro study and did not include any human and animal participants. This step ensured that all ethical guidelines were adhered to throughout the research process.

## Results

The results of this study are organized to provide a clear understanding of the comparative performance of different RPD frameworks, emphasizing both statistical significance and practical implications.

Maximum load comparison

**Figure 3:**
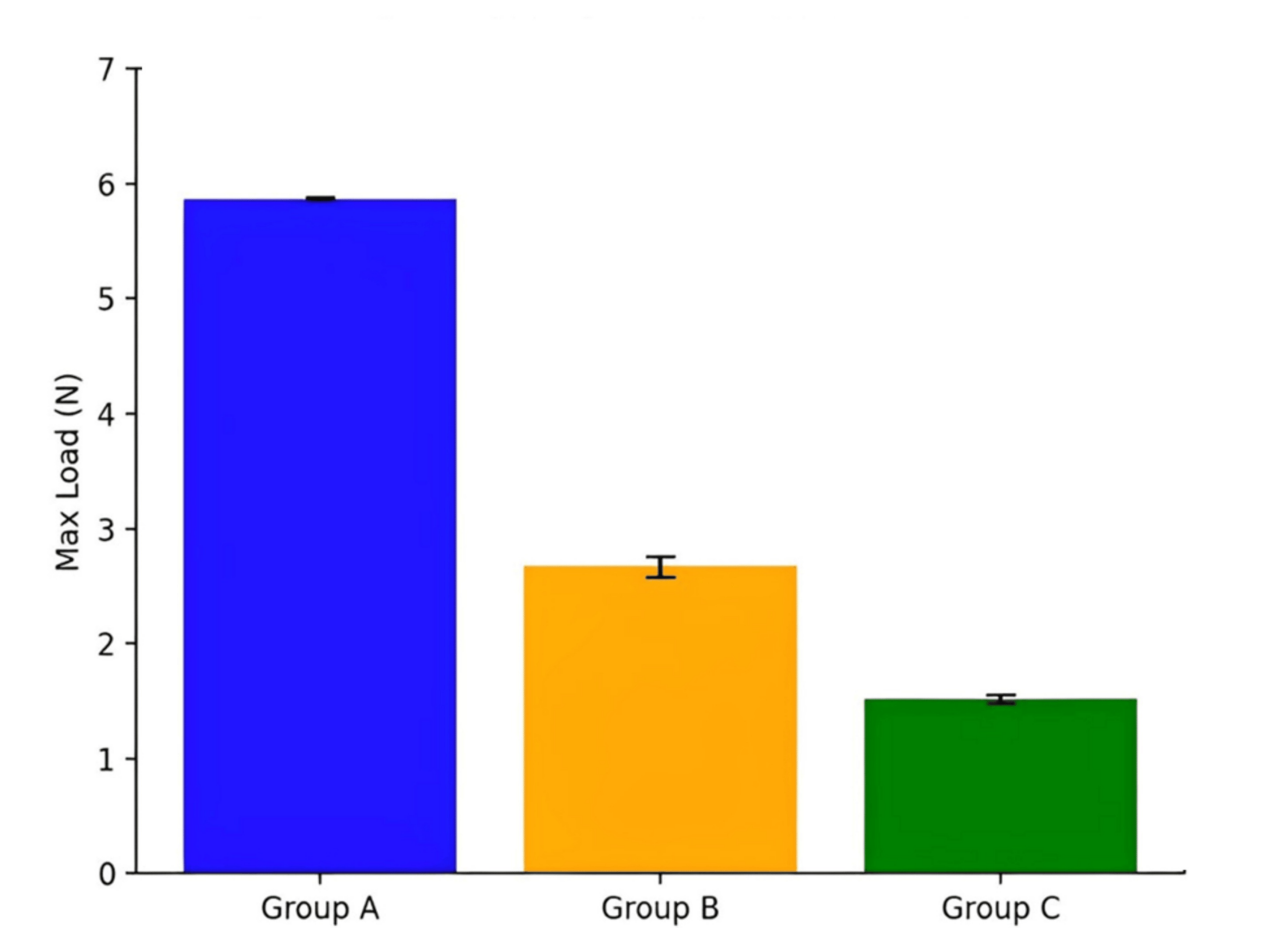
Comparison of maximum load among PEEK, acetal, and Co-Cr groups, illustrating significant differences and their clinical implications. PEEK: polyetheretherketone; Co-Cr: cobalt-chromium

The maximum load capacity of the three framework groups is illustrated in Figure 3 and Table 1. Group A (PEEK) demonstrated a significantly higher maximum load (5.87 ± 0.01 Newtons) compared to Group B (acetal) at 2.67 ± 0.09 Newtons and Group C (Co-Cr) at 1.52 ± 0.04 Newtons. Statistical analysis using an independent T-test revealed substantial differences among the groups, with a significance level of P < 0.001.

**Table 1:** Comparison of the maximum load between the three groups. * Significant difference between groups (P < 0.001).

Groups	Max. Load (N)	Mean Difference	95% Confidence Interval	Independent T-test	Sig. (Two-Tailed)
Group A	5.87 ± 0.01	-	5.75-5.95	12.34	<0.001*
Group B	2.67 ± 0.09	3.20	2.55-2.78	9.21	0.003*
Group C	1.52 ± 0.04	1.15	1.48-1.56	8.45	0.002*

The superior load-bearing capability of PEEK suggests its potential for clinical applications where higher functional forces are required, such as cases involving heavy occlusal loads or extended distal extensions. These findings underscore PEEK's durability advantage and ability to maintain stability under stress compared to acetal and Co-Cr frameworks.

Descriptive statistics for retentive force

**Figure 4:**
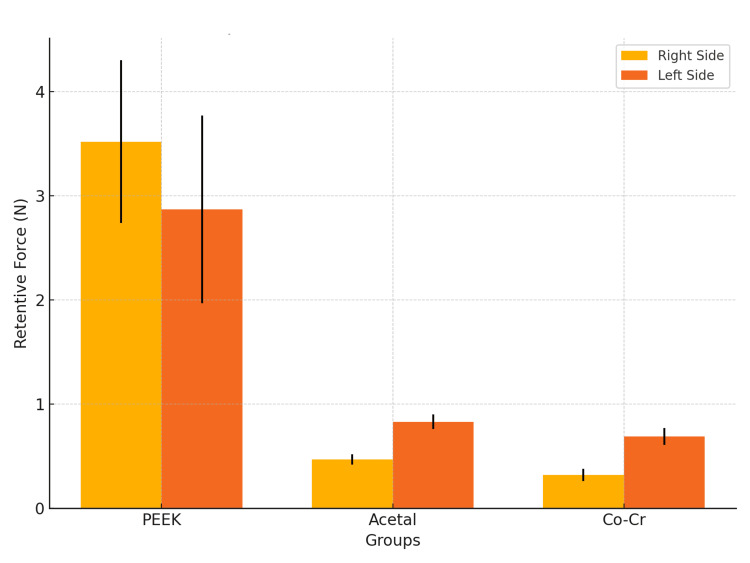
Descriptive statistics for retentive forces of PEEK, acetal, and Co-Cr groups, measured separately for the right and left sides. PEEK: polyetheretherketone; Co-Cr: cobalt-chromium

The retentive force for each group, measured separately for the right and left sides, is depicted in Figure 4 and Table 2. This approach was used to assess symmetry in retention and to identify material-specific performance differences under varied conditions.

**Table 2:** Descriptive statistics of the retentive forces in each of the tested groups. PEEK: polyetheretherketone; Co-Cr: cobalt-chromium

Groups	Sides	Retentive Force (N)	Min	Max	Mean	SD	95% Confidence Interval
							Lower
PEEK	Right	15	2.35	4.67	3.52	0.78	0.97
	Left		1.98	3.64	2.87	0.90	0.99
Acetal	Right	15	0.32	0.56	0.47	0.02	0.34
	Left		0.78	0.98	0.83	0.52	0.27
Co-Cr	Right	15	0.27	0.49	0.32	0.12	0.19
	Left		0.57	0.85	0.69	0.09	0.29

PEEK Group

Retentive forces ranged from 2.35 to 4.67 Newtons (mean: 3.52 ± 0.78 N) on the right side and from 1.98 to 3.64 Newtons (mean: 2.87 ± 0.90 N) on the left side.

Acetal Group

Retentive forces were lower, ranging from 0.32 to 0.56 Newtons (mean: 0.47 ± 0.02 N) on the right side and 0.78 to 0.98 Newtons (mean: 0.83 ± 0.52 N) on the left side.

Co-Cr Group

Demonstrated the lowest values, with right-side measurements of 0.27 to 0.49 Newtons (mean: 0.32 ± 0.12 N) and left-side measurements of 0.57 to 0.85 Newtons (mean: 0.69 ± 0.09 N).

These results indicate that PEEK provides superior retention, which could improve the functional stability and comfort of RPDs, especially in clinical cases requiring higher retention forces.

Comparative analysis of retentive force

**Figure 5:**
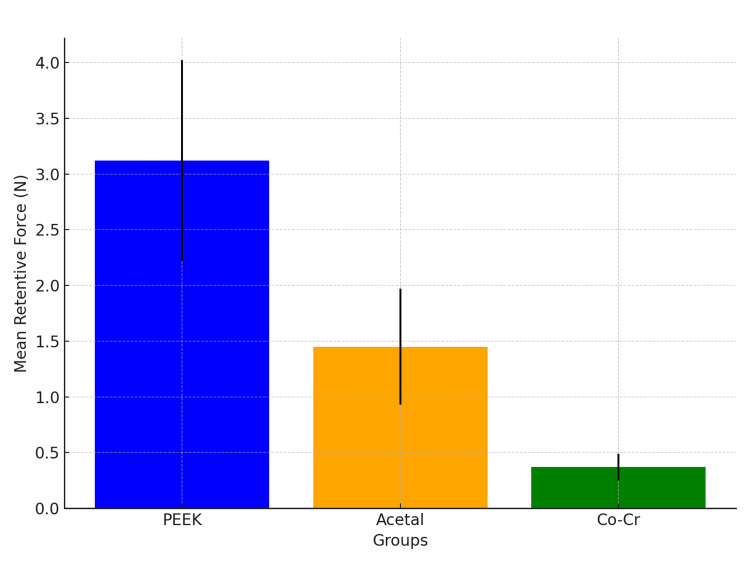
Comparative analysis of retentive forces among PEEK, acetal, and Co-Cr groups, highlighting significant differences and practical implications. PEEK: polyetheretherketone; Co-Cr: cobalt-chromium

The comparative analysis of retentive forces is presented in Figure 5 and Table 3. Statistical analysis revealed significant differences across all materials (P < 0.001). The PEEK group consistently exhibited higher retentive forces than acetal and Co-Cr on both sides.

**Table 3:** Comparison of PEEK, acetal, and Co-Cr on the right and left sides. PEEK: polyetheretherketone; Co-Cr: cobalt-chromium

Material	Right Side Mean ± SD (N)	Left Side Mean ± SD (N)	Mean Difference (N)	Confidence Interval	Independent T-test	P-value
PEEK	3.52 ± 0.90	2.87 ± 0.148	3.12	2.55-5.95	21.78	0.001
Acetal	0.47 ± 0.02	0.83 ± 0.52	1.45	0.34-2.78	33.69	0.001
Co-Cr	0.32 ± 0.12	0.69 ± 0.09	0.37	0.19-1.56	15.24	0.004

Interestingly, the right side showed higher retention across all groups compared to the left side, suggesting potential asymmetry in force distribution. For clinicians, this highlights the importance of accounting for material properties and clasp design in optimizing retention for different patient needs.

## Discussion

Regardless of the type of clasp employed, an RPD can be effectively retained as long as the force required to bend the clasps over the largest bulges of the teeth exceeds the force attempting to dislodge the denture [16,17]. The variations in retention forces observed in these tests may be attributed to discrepancies in the proportions of the wax patterns used for clasp fabrication, the degree of deflection, the flexibility of the clasps, and the characteristics of the testing model [11,18].

Thermoplastic clasps can achieve satisfactory retention even when their dimensions differ from those of traditional metal clasps [12-16]. In some instances, a thicker thermoplastic clasp may be necessary to engage deeper undercuts effectively [17,18]. Our findings indicate that acetal (Group II) and Co-Cr (Group III) clasps exhibited significantly lower retentive forces compared to PEEK clasps (Group I). Previous research suggests that the ideal retention force for partial removable dentures ranges from 3 to 7.5 N [11], with another study identifying 5 N as adequate for ensuring sufficient retention [16,19].

Acetal resin demonstrates a notable proportional limit and minimal viscous flow, enabling it to exhibit elastic behavior over a sufficiently wide range, thus making it suitable for clasp production [19]. Other studies corroborate our findings regarding the retention capabilities of PEEK and acetal clasps. Specifically, clasps constructed from 3 mm thick PEEK material exhibited significantly greater retention forces when compared to acetal clasps. The loss of clasp retention observed during fatigue resistance testing serves as a reliable indicator of permanent deformation [20-22].

Moreover, another investigation reported that titanium clasps exhibited the highest average retentive force, followed by PEEK clasps, whereas acetal resin clasps displayed the lowest average retentive force. The reduced retentive strength of acetal clasps can be attributed to their increased flexibility [23].

El-Segai and Abbas determined that innovative clasps made from PEEK material exhibited significantly greater retention forces compared to acetal clasps. Their findings also indicated that the loss of retention resulting from fatigue resistance testing reliably reflected permanent deformation [20]. Supporting this, Fayyad and Helmy noted that PEEK demonstrated superior retention compared to various other thermoplastic materials [21]. Our results further indicated that the acetal group had the lowest mean retention value compared to PEEK, with significant differences attributed to PEEK’s high impact strength [21].

Furthermore, a comprehensive analysis established that the retentive forces of PEEK clasps are greater than those of acetal resin. This enhanced strength in PEEK, relative to the other investigated resins, may be attributed to its unique composition, including the presence of an inorganic filler and its semicrystalline nature (30-35% crystalline), which likely enhances stiffness compared to acetal, which lacks this ceramic filler. A study corroborating our findings assessed the primary load required to bend acetal resin clasps, revealing minimal load requirements when deflection reached 0.5 mm, indicating a high degree of flexibility [21].

The observed outcomes may also stem from the increased erosion of PEEK in comparison to Co-Cr alloys. The findings of this investigation align with those of Harb et al. [22], who reported a statistically insignificant decline in retention for both groups after the second three months of the study. However, they noted greater retention loss in the PEEK group relative to the metal group. This discovery is consistent with the findings of Elsayed et al. [24] and Rexhepi et al. [25], who similarly observed a decline in retention of Co-Cr clasps during simulation tests, attributing the decrease in retention force to permanent deformation of the metal.

While our study provides valuable insights into the retention capabilities of different clasp materials, it is not without limitations. The sample size for each group may limit the generalizability of the findings, and further studies with larger cohorts are necessary to validate these results. Additionally, the in vitro nature of the testing may not fully replicate the complex oral environment, which includes factors such as salivary composition, oral hygiene practices, and the dynamic nature of occlusal forces that can influence clasp retention. Future research should aim to address these limitations by conducting longitudinal clinical studies to assess the real-world performance of these materials over time.

## Conclusions

This study demonstrated that PEEK frameworks outperformed acetal and Co-Cr in terms of retentive forces, highlighting its potential as a preferred material for RPD frameworks. These findings offer clinicians evidence-based guidance for material selection based on case-specific needs, such as aesthetic requirements, durability, and patient preferences.

Further research is necessary to address the translation of in vitro findings into clinical practice. Future studies could explore the long-term performance of these materials in diverse patient populations, considering factors such as aging, dynamic oral forces, and patient feedback. Additionally, advancing CAD/CAM technology and material innovations may provide new avenues for improving the design and functionality of RPD frameworks.
